# Supplementary material for: Content-rich biological network constructed by mining PubMed abstracts
Source: BMC Bioinformatics. 2004 Oct 8;5:147. doi: 10.1186/1471-2105-5-147 (PMC528731; doi:10.1186/1471-2105-5-147)
Supplement: Additional File 5 — The original Chilibot query results of the term "long-term potentiation (LTP)" and 22 other terms, limiting the latest references analyzed to the years 1990, 1995, 2000, and 2004. [file 1471-2105-5-147-S5.bz2 › chilibotAdditionalFile5/ltp2004/html/TAU.html]

 


**TAU** (Input: TAU ) 

---


**Google Searches:** Entire Web  | EDU domain only  | PDF files only

.

**External Links:** OMIM |
LocusLink
| Swissprot
| GeneCards

  
**Maps of TAU**

Simple
Complete
graph in
radiant
tree
square
layout.

**New Hypothesis !**

**Synonyms** 

- tau   [PubMed]

**Synopsis**

- These results suggest that in cells that express **tau**, receptors coupled to cytosolic phospholipase A2 may activate PLC gamma isozymes indirectly in the absence of tyrosine phosphorylation through the hydrolysis of phosphatidylcholine to generate AA.  J Biol Chem, 1996 
  [20]

- p25 Cdk5 shows higher phosphorylating activity to **tau** than p35 Cdk5 in a phosphorylation site specific manner.  NeurosignalsNeurosignals, 1996 
  [19]

- Wehave recently shown that phospholipase C gamma PLC gamma is activated by **tau**, a neuronal cell specific microtubule associated protein, in the presence of arachidonic acid.  J Biol Chem, 1999 
  [19]

- Antibodies against microtubule associated protein **Tau**, which is expressed in developing neurites, showed that T3 enhanced neurite formation.  Neuroreport, 1992 
  [19]

- Furthermore, MAP 2 and **tau** protein showed a development dependent change in expression from the juvenile toward the adult form.  J Neurochem, 1992 
  [19]

- The enzyme phosphorylated smooth muscle myosin light chain, synapsin I, microtubule associated protein 2, **tau** protein, myelin basic protein, histone H1, and tyrosine hydroxylase in a calcium calmodulin dependent manner, suggesting that the enzyme is a multifunctional calmodulin dependent protein kinase capable of phosphorylating a large number of substrates.  J Biol Chem, 1992 
  [16]

- In addition, using permeabilized lymphoma cells, we have found that 1 GTP or GTP **tau** S augments, and pertussis toxin inhibits, phospholipase C PLC activity and receptor capping.  J Immunol, 1990 
  [16]

- During postnatal development, the intensity of the phosphate dependent staining decreased, suggesting that phosphorylation of **tau** proteins in perikarya and dendrites may be essential for early steps in neuronal morphogenesis during cat cerebellum development.  Brain Res Bull, 1994 
  [16]

- These studies indicate that **tau** associates, either directly or indirectly, with PLC gamma in situ, suggesting that **tau** may be appropriately localized to participate in the regulation of signal transduction through the PLC gamma pathway in vivo.  Neuroreport, 1998 
  [16]

- Phalloidin staining and immunohistochemistry showed that the neuroblast was richer in F actin, beta tubulin, MAP1, MAP2, **tau**, calspectin, and synapsin I than the matrix cell.  Arch Histol Cytol, 1992 
  [15]

- We were unable to obtain any morphological indication of neurodegenerative processes in these animals.We suggest that the moderate increase in **tau** protein and phosphorylation may be part of the neuroprotective mechanism.  Neuroscience, 2001 
  [15]

- These findings suggest that PLC **tau**, and perhaps the 76 kDa co precipitated protein, are substrates of cyclic AMP dependent protein kinase in BALB c 3T3 cells however, the lack of effect of cyclic AMP elevation on PDGF stimulated inositol phosphate formation indicates that the intrinsic activity of PLC **tau** is unaltered by cyclic AMP mediated phosphorylation.  Biochem J, 1990 
  [13]

- The time constant of decay **tau** calculated from pooled data for each group yielded declines in PS amplitude by 63% in 17.4 days in controls and 13.4 days in Pb exposed animals.  Brain Res, 1998 
  [11]

- GSK 3 is implicated in the regulation of many physiological responses in mammalian cells by phosphorylating substrates including neuronal cell adhesion molecule, neurofilaments, synapsin I, and **tau**.  Biochem Biophys Res Commun, 1998 
  [10]

- Cholinergic neurons are the main pharmacologic target but there are many other ones GABA ergic system, **Tau** protein, amyloid.  EncephaleEncephale, 1998 
  [10]
